# Investigation of Multi-Factor Stress Corrosion Cracking Failure of Safe-End Feedwater Lines of Submarine Power System

**DOI:** 10.3390/ma17061381

**Published:** 2024-03-18

**Authors:** Chenlong Ji, Zhongliang Zheng, Ziming Qin, Hao Xue

**Affiliations:** 1 Naval Equipment Department of PLA, Beijing 100841, China; chenlongji@163.com (C.J.); zhongliangzheng@163.com (Z.Z.); zimingqin@163.com (Z.Q.); 2 Shenyang National Laboratory for Materials Science, Northeastern University, 3-11 Wenhua Road, Shenyang 110819, China

**Keywords:** stress corrosion cracking, safe-end feedwater lines, galvanic corrosion, residual stress, vortex flow

## Abstract

The corrosion process under the complex safe-end feedwater line conditions was investigated via experimental lab testing and numerical simulation. The corrosion of safe-end feedwater lines was controlled through the combination of galvanic corrosion, residual stress, and flow velocity. Firstly, galvanic corrosion occurred once the 20 steel was welded with 316L stainless steel. The pitting corrosion could be observed on the 20 steel side of the weld joint. Secondly, a vortex flow was detected around the welding bump and within the pits. The growth of the pits was accelerated in both the vertical and horizontal directions. Finally, under the residual stress condition, the stress intensity factor (*K*) at the bottom of the pits was easier to reach than the critical stress intensity factor (*K*_ISCC_). Then, pitting was transformed into stress corrosion cracking which then propagated along the weld line. Therefore, the critical factor inducing the failure of safe-end feedwater lines was the combined action of galvanic corrosion, residual stress, and flow velocity.

## 1. Introduction

In recent years, increasing attention has been paid to the development of deep-sea resources around the world. Being a country rich in Marine resources, China has actively promoted deep-sea exploration and research. In deep-sea exploration, the safety of special boiler systems as the main sources of power and heat energy is of great significance for the various deep-sea operations and scientific works. Under specific submarine conditions, the boiler systems may undergo failure due to continuous high-temperature and high-pressure exposures [1,2,3].

**Figure 1:**
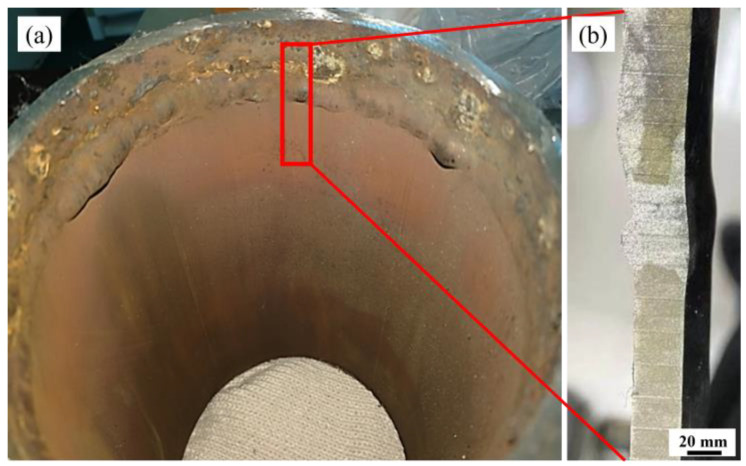
The basic macro morphology of safe-end feedwater lines: (**a**) the macro morphology of lines; (**b**) the cross-sectional morphology of the weld.

While the whole steam boiler is made of 20 steel and the steam pipeline is composed of 316L stainless steel, the safe-end feedwater line is welded with both kinds of steel [4,5,6]. In the field investigation, under abnormal working conditions, leakage caused by corrosion at the weld of the safe-end feedwater lines often leads to equipment failure, as shown in Figure 1. The weld corrosion failure is usually provoked by the potential difference between various materials after the liquid film covers the weld. In general, galvanic corrosion between two metals occurs when the potential difference (ΔE) is greater than 50 mV and becomes severe at ΔE > 250 mV [7,8,9,10,11,12,13].

However, because the liquid in the boiler pipeline experiences a high-pressure environment, the flow also makes the corrosion process more intense. Numerous studies have shown that the rapid fluid has an adverse effect on the deposition of products at the interface and the presence of bumps around the weld leads to the formation of local turbulence when the liquid flows through [14,15,16]. It not only enhances the erosion and corrosion of the pipeline [17,18], but also further alters the microscopic morphology of the interface owing to the change of the corrosion scale. In addition, under the influence of varying flow forms and flow-induced wall shear stress, the deposition rate and dissolution mechanism of the product film on the substrate surface also become more complex [19,20,21]. Therefore, the synergistic effect of electrochemical corrosion and fluid mechanical erosion on the development of corrosion is noticeable. In addition, due to the defects promoted by the uneven heating upon the welding itself, residual stress inevitably exists near the weld. As a germination factor causing pitting corrosion, residual stress will rapidly expand into cracks in harsh environments, enhancing the SCC sensitivity of the pipeline steel [22,23,24,25]. Additionally, similar point corrosion (SCC) caused by residual stress and its derivative cracks was also found in the failed pipelines. In a word, the failure arising in the weld of a safety feed pipe may be the result of many factors, including galvanic corrosion under the synergistic effect of high temperature, high pressure, residual stress, and fluid erosion. Meanwhile, the influence of coupling between fluid and residual stress of welded joints in similar corrosive media on galvanic corrosion has not yet been reported.

This article systematically studies the mechanism of galvanic corrosion in complex environments so as to elucidate the causes of corrosion failures in the safe-end feedwater of the power systems and promote the development of the power industry. In particular, the corrosion failures of the welded joints were investigated through electrochemical testing, immersion testing, residual stress testing, and critical stress intensity factor assessment for stress corrosion cracking (*K*_ISCC_). The experimental results were then compared with the Fluent model simulation data.

## 2. Experimental Section

### 2.1. Materials and Solution

The chemical composition of 20 steel and 316L stainless steel used in this work is shown in Table 1. An in-site 20 steel/316L welding wire/316L pipeline sample was applied in the investigation. Before the experiments, the measuring section of the sample tube was ground with a 2000 grit abrasive paper along the tensile direction, then degreased with ethanol, washed with distilled water, and, finally, dried.

The contact solution of the simulated water supply pipeline was employed as an experimental solution under the environmental conditions described in Table 2. The water quality was classified into normal water and the deviated one. The normal water quality corresponded to the actual operating condition. Moreover, to evaluate the corrosion behavior effectively, an extreme deviation water solution was designed using analytical reagent chemicals and deionized water (the resistivity of 18 MΩ/cm); this condition exists in in-site conditions and leads to more severe corrosion of safe-end feedwater lines. After in-site measuring, the deviation water quality condition was identified.

### 2.2. Electrochemical and Immersion Testing

**Figure 2:**
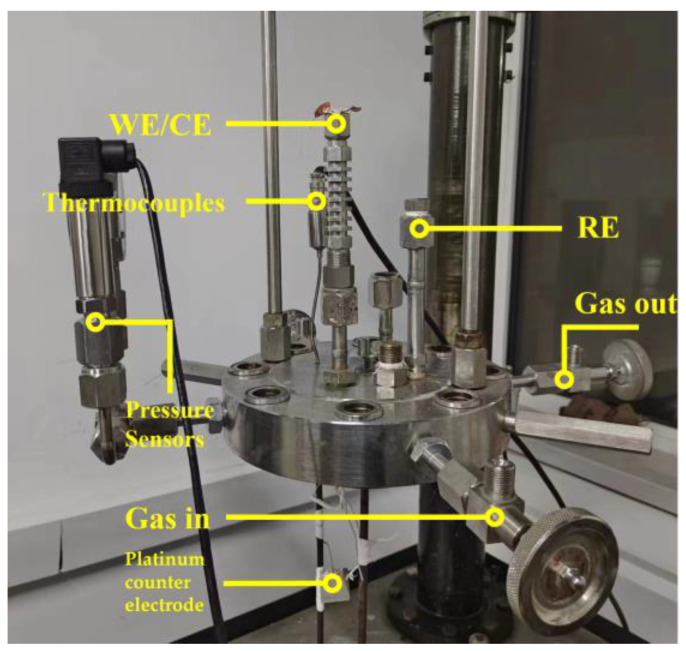
Schematic diagram of electrochemical autoclave and electrode location.

The metal corrosion potential (*E*_corr_) and galvanic corrosion tests were carried out in a high-temperature and high-pressure reactor at a design temperature of 25–200 °C and a pressure of 0.1–25 MPa according to the schematic shown in Figure 2. A three-electrode system consisting of a working electrode, a platinum counter electrode, and an external pressure-balanced Ag/AgCl reference electrode (TOSHIN, UHP, Tokyo, Japan) was employed. The above two electrodes were connected outside the high-temperature and high-pressure electrochemical reactor. The cell was then connected to the electrochemical workstation for further measurements of the corrosion potentials and currents. The open circuit potentials of 20 steel and 316L steel were acquired over 240 h at 100 °C. The Ag/AgCl reference electrode was subjected to high-temperature and high-pressure conditions, and its potential was corrected with respect to the temperature as follows [26,27,28]:

(1) $$\begin{array}{c} E_{SHE}=E_{obs}+0.2866-0.001\left(T-T_{0}\right)+\\ 1.754\times 10^{-7}{\left(T-T_{0}\right)}^{2}-3.0\times 10^{-9}{\left(T-T_{0}\right)}^{3} \end{array}$$

where *E*_SHE_ refers to the potential relative to the standard hydrogen electrode, *E*_obs_ is the potential measured in an extreme environment, *T* denotes the testing temperature (°C), and *T*_0_ is room temperature (25 °C).

The immersion tests in the solution were carried out in the high-temperature and high-pressure dynamic stainless autoclave containing the rotating cage. Each set of experimental data was analyzed and characterized using three parallel specimens. The environmental parameters kept during the immersion tests are listed in Table 2. The rotation speed was controlled at 5π rad/s, and the corresponding sample surface flow rate was 0.5 m/s. The conditions set upon the electrochemical and immersion tests are provided in Table 2.

The corrosion rate ($V_{corr},\;in\;mm/yr$) was calculated as follows:

(2) $$V_{corr}=\frac{87,600\Delta g}{\rho tS}$$

where Δg denotes the weight loss of samples, g; *ρ* is the sample density, g/cm^3^; *t* refers to the experimental time, h; and *S* is the area of the sample surface, cm^2^.

### 2.3. Fluent Model Simulation

**Figure 3:**
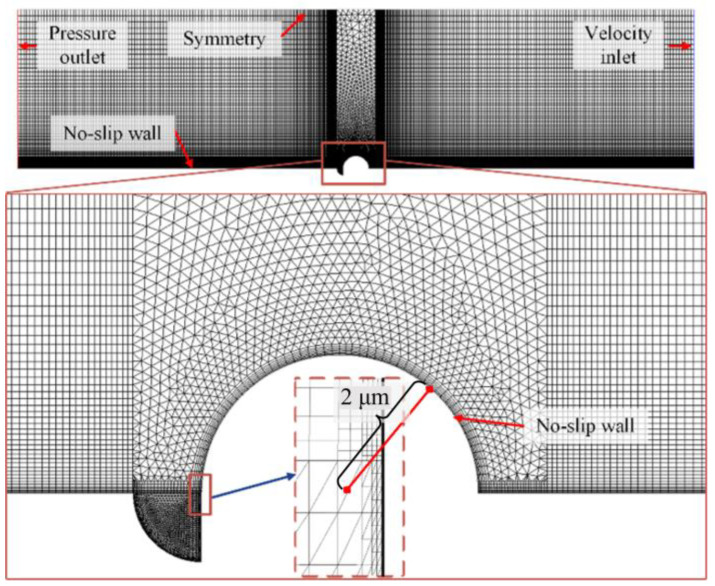
Geometric model and mesh scheme of pitting and welding bumps.

Numerical simulation of the flow field in the model was conducted using a commercial computational fluid dynamics (CFD) package (Fluent 2021 R1), based on the finite volume approach. The geometric model and mesh scheme of pits and the area around the welding bump are shown in Figure 3. According to the statistical results, the pitting depth was set as 2 μm and a semicircle of 4 μm was selected as the representative welding bump. A total of 18,886 elements were generated in the simulation. Since the flow near the wall was in the laminar boundary layer region, the laminar transfer model was used for deducing the respective equations. The inlet velocity of water was 0.5 m/s. The Fanning friction factor was a function of Reynolds number (Re, being equal to 64,000 in these iterative calculations), and SIMPLE (semi-implicit method for pressure-linked equations) algorithms were used to solve the momentum equations, whereby the pressure term was modified in the discretized momentum equation (N-S equation) to renew the velocity and pressure fields simultaneously. The iterative calculations of primitive variables, such as flow velocity, were terminated as soon as the residual norm criteria of 10^−6^ was reached. Each set of experimental data was analyzed and characterized using three parallel specimens.

### 2.4. Residual Stress Testing

An X-ray diffractometer (XRD) was integrated into an X-ray stress meter to measure the crystal plane spacing in the welded joint sample of the pipe section, as well as to determine the macro strain and the welding residual stress of the sample according to Hooke’s law. The measurements were conducted while conforming to GB/T7704-2017 standard [29]. The evaluation of welding joint stress consisted of establishing the axial residual stress of the weld on the outer surface of the sample pipe.

### 2.5. Slow Strain Rate Test

Before the testing, the slow strain rate test (SSRT) specimens were immersed in the experimental environment for 12 h. As a working electrode, the CT specimens used in the electrochemical measurements were shielded by 705 silicone except for the gauge area. The tensile specimens were welded on platinum wire and shielded by heat-shrinkable polytetrafluoroethylene on the tube to avoid galvanic corrosion. The loading holes of SSRT specimens were insulated from the loading column by ceramic packing. After immersion, the SSRTs were performed immediately with a strain rate of 10^−6^ s^−1^. The SSRTs were conducted in conditions of normal operation water quality and deviation water quality conditions. Each test was reproduced three times to confirm the reproducibility of the data.

### 2.6. Critical Stress Intensity Factor for Stress Corrosion Cracking (K_ISCC_)

The direct current potential drop (DCPD) experiment of crack propagation within 20 steel/316L weld joints was also conducted in an HTHP-SCC electrochemical system at the normal (105 °C) and deviated (180 °C) water qualities, using a 12.7 mm thick compact tensile (CT) specimen with a pre-crack [30,31,32,33,34]. The specimen was insulated from the solution by a high-temperature epoxy coating (2 μm) except for cracking and measurement points. A constant displacement load was applied during the crack propagation process. The initial stress intensity factors (*K*) were set as 29 MPa·m^1/2^ and 28 MPa·m^1/2^, corresponding to the normal operation water quality and deviation water quality, respectively. The value of *K* was measured until the crack stopped propagating. The final value of *K* was *K*_ISCC_. Each set of experimental data was analyzed and characterized using three parallel specimens.

The change of the deepest crack *K*_1_ in the pipeline with pitting depth under a certain axial stress was evaluated using the formula below:

(3) $$K_{Ⅰ}=\sigma_{t}\sqrt{\frac{\pi a}{Q}}F$$

In this formula, $\sigma_{t}$ is the pipeline axial stress, $Q=1+1.464{\left(\frac{a}{l}\right)}^{1.65}$, $F=1+(0.02+\alpha \left(0.0103+0.00617\alpha \right)+0.0035(1+0.7\alpha )(\frac{R}{t}-5{)}^{0.7})Q^{2}$, $\alpha =\frac{a}{t}\cdot \frac{a}{2l}$.

## 3. Result and Discussion: From Normal to Deviation Water Quality

### 3.1. The Galvanic Corrosion of 20 Steel and 316L

**Figure 4:**
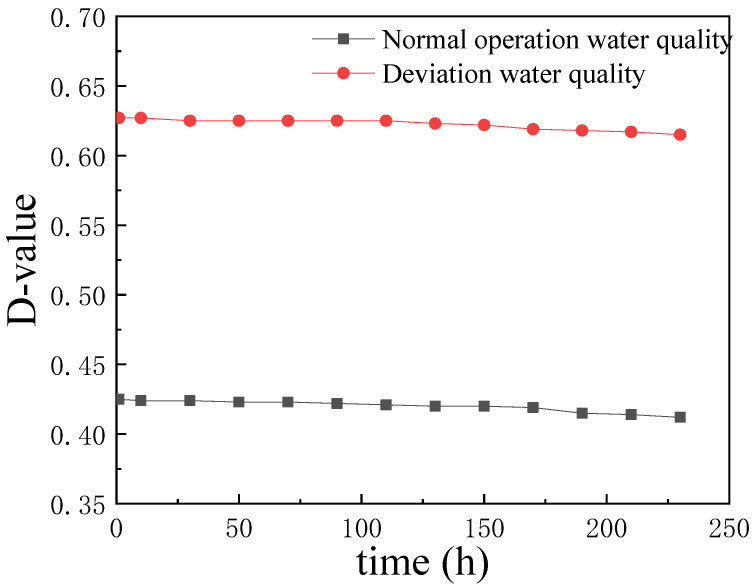
The corrosion potential (*E*_corr_) difference of 20 steel/316L.

The corrosion potential (*E*_corr_) tests of 20 steel and 316L stainless steel were carried out in a high-temperature (105 °C for normal operation water quality and 180 °C for deviation water quality) and high-pressure electrochemical system [35]. As shown in Figure 4, the potential difference between 20 steel and 316L steel samples was 0.411 ± 0.015 V in the case of the normal operation water quality and 0.605 ± 0.023 V for the deviation water quality. Thus, the galvanic corrosion occurred in the 20 steel/316L weld couple, in which 20 steel and 316L steel served as anode and cathode, respectively, and H^+^ was the oxidant. The influence of the galvanic couple on the corrosion rate was investigated by performing an immersion test. The corrosion rates of a single 20 steel sample and 20 steel in the weld were measured in the normal and deviation water quality environments, as shown in Table 3. Under the criterion of the normal operating water quality, the 20 steel exhibited a lower corrosion rate, while that of 20 steel in the weld couple increased, resulting from the galvanic effect. As for the deviation water quality, the corrosion rate increased by an order of magnitude compared with the normal operating water quality. The increase in corrosion rate for 20 steel in the weld couple under deviation water quality conditions was due to the greater potential difference. Thus, the galvanic effect significantly promotes the corrosion of 20 steel.

### 3.2. Flow Results of the Safe-End Feedwater Lines

**Figure 5:**
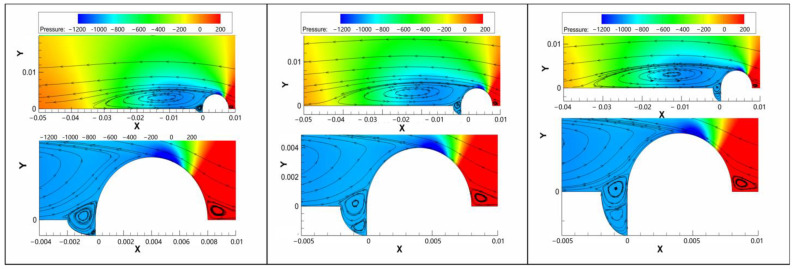
The pressure field around the pitting in the weld area (here, X is the pressure (MPa), and Y is the pitting dimension (μm)).

The pressure field around the pitting within the 316L weld area is shown in Figure 5. The surface profile significantly shifted to a roughness state. The vortex flow in the pitting area exhibited a lower pressure compared with the laminar flow above the pitting area. The pressure difference could promote mass exchange between the pitting area and the above laminar flow.

**Figure 6:**
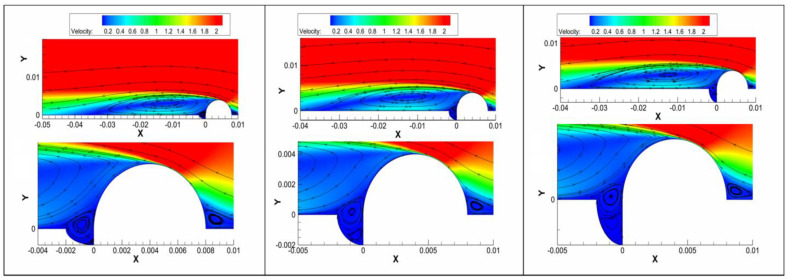
The flow velocity field around the pitting in the weld area (here, X is the flow velocity (m/s), and Y is the pitting dimension (μm)).

A significant variation of flow velocity across the turbulent vortex was observed within the pitting area in Figure 6. With the increasing pitting depth, the turbulent vortex gradually penetrated the bottom of the pitting, even though the flow velocity decreased significantly.

### 3.3. Residual Stress and SCC Susceptibility

To explore the consistency of internal stress after welding and its effect on the corrosion resistance of the materials, the 20 steel/316L welding wire/316L specimens cut from the site were tested near the wire/316L interface, and the respective results are given in Table 4. The measured residual stress values revealed tensile stress at the inner surface and compressive stress at the outer surface of the pipeline. The mechanical deformation caused by tensile stress can promote the redistribution of heterogeneous phases in electrochemical reactions, leading to an increase in anode current density, followed by enlargement in the cathode reaction area, and thereby accelerating corrosion and enhancing the potential risk of SCC occurrence.

**Figure 7:**
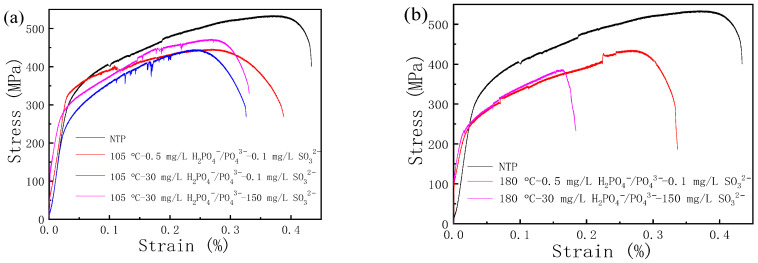
Stress–strain curves of 20 steel at (**a**) 105 °C; and (**b**) 180 °C upon slow strain rate tensile tests in H_2_PO_4_^−^/PO_4_^3−^ and SO_3_^2−^ solutions.

The stress–strain curves of 20 steel at 105 °C and 180 °C are depicted in Figure 7. Compared with the normal temperature and pressure (NTP) nitrogen environment, there was an overall decrease in the stress−strain curves of 20 steel in H_2_PO_4_^−^/PO_4_^3−^ and SO_3_^2−^ solution environments at 105 °C, both in terms of intensity and range. The electrolyte environment was maintained with NaH_2_PO_4_ and Na_2_SO_3_. In particular, at the SO_3_^2−^ concentration of 0.1 mL/L, the stress−strain curves (Figure 7a) in 0.5 mL/L and 30 mL/L H_2_PO_4_^−^/PO_4_^3−^ decreased compared with those in the NTP environment. Once the concentration of H_2_PO_4_^−^/PO_4_^3−^ increased from 0.5 to 30 mL/L, the curve continued to decline, and the yield strength decreased significantly. At the H_2_PO_4_^−^/PO_4_^3−^ concentration of 30 mL/L and the SO_3_^2−^ concentrations from 0.1 to 150 mL/L, the tensile curve moved upward as a whole, and fracture occurred at a higher stress level. At the temperature of 180 °C, the stress−strain curve of 20 steel in an H_2_PO_4_^−^/PO_4_^3−^ and SO_3_^2−^ solution environment dropped compared with that in the NTP environment, as did the shape variable at fracture, meaning a decrease in yield strength and tensile strength. As soon as there was the increase in H_2_PO_4_^−^/PO_4_^3−^ and SO_3_^2−^ concentrations (Figure 7b), the stress–strain curves exhibited a slightly increasing trend and the sample breaks at a lower strain at break.

**Figure 8:**
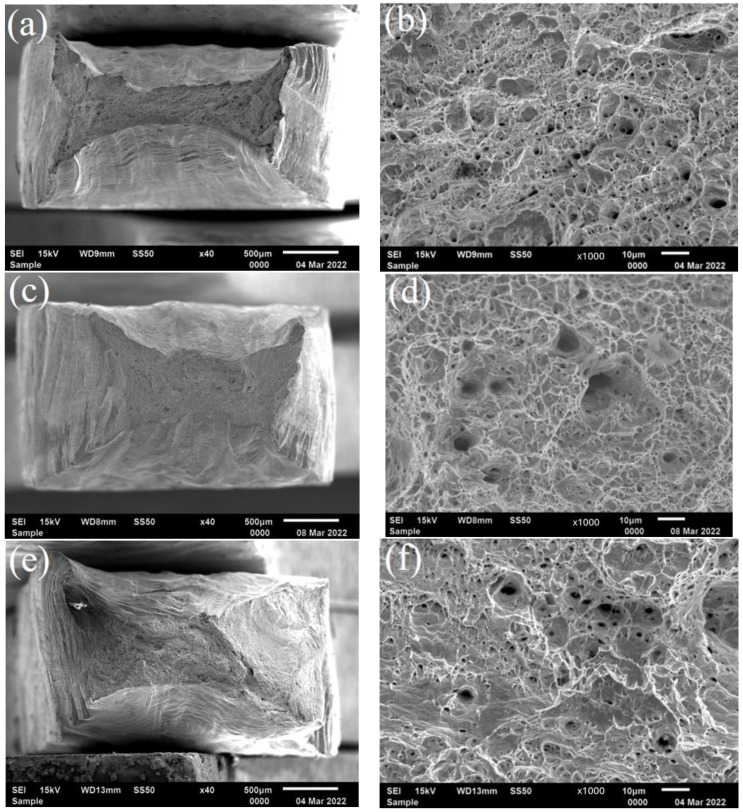

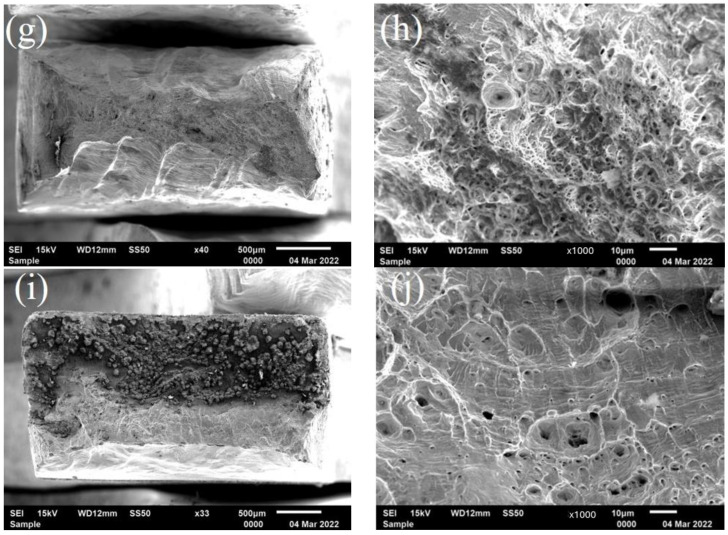
Fracture morphology of the weld area during slow strain rate tensile tests in a corrosive environment. (**a**,**b**) 105 °C—0.1 mg H_2_PO_4_^−^/PO_4_^3−^ (1:1)—0.5 mg SO_3_^2−^; (**c**,**d**) 105 °C—30 mg H_2_PO_4_^−^/PO_4_^3−^ (1:1)—0.5 mg SO_3_^2−^; (**e**,**f**) 105 °C—30 mg H_2_PO_4_^−^/PO_4_^3−^ (1:1)—150 mg SO_3_^2−^; (**g**,**h**) 180 °C—0.1 mg H_2_PO_4_^−^/PO_4_^3−^ (1:1)—0.5 mg SO_3_^2−^; (**i**,**j**) 180 °C—30 mg H_2_PO_4_^−^/PO_4_^3−^ (1:1)—150 mg SO_3_^2−^.

Figure 8 displays the fracture morphology of 20 steel at 105 °C at the 0.1 mg H_2_PO_4_^−^/PO_4_^3−^ and 0.5 mg SO_3_^2−^ concentrations. As shown in Figure 8a–f, with the increasing H_2_PO_4_^−^/PO_4_^3−^ (1:1) and SO_3_^2−^ concentrations in the solution, the neck shrinkage of the fracture became less pronounced, resulting in a larger dimple, in which more and more quasi-cleavage surfaces were observed. Moreover, the toughness of the material gradually decreased, indicating that severe stress corrosion occurred in the weld. At the temperature of 180 °C (Figure 8g,i), the neck shrinkage of the fracture in the weld zone almost disappears compared with the fracture morphology at 105 °C. It can be observed from Figure 8h,j that the number of dimples dropped to a large extent, the dissociated surface proportion significantly increased, and the fracture surface was relatively flat, showing obvious brittle fracture characteristics.

In this experiment, a plate sample of 20 steel/316L welded joints was first prepared. The constant displacement loading was carried out to promote the crack growth, and the crack growth speed and change in stress intensity factor (*K*) were measured through DCPD monitoring of resistance until the cracks stopped growing to obtain the critical stress intensity factor (*K*_ISCC_) for stress corrosion cracking (the threshold value for judging the occurrence of stress corrosion cracking). As for the opening mode crack, the stress intensity factor (*K*_I_) of the CT specimen was calculated as follows:

(4) $$K_{I}=\frac{YP}{B\sqrt{W}}$$

(5) Y=2+aW1 − a3W0.886+4.64aW−13.32aW2+14.72aW3−5.6aW4

where *P* is the loading force, *B* represents the effective thickness of the sample, *a* is the crack depth, and *W* denotes the effective width of the sample.

**Figure 9:**
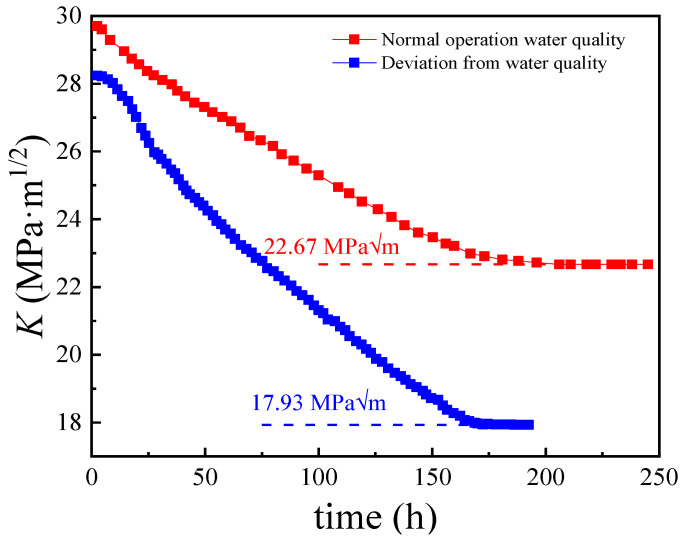
*K*_ISCC_ of 20 steel/316L weld joints measured by DCPD under normal operation water quality and deviation water quality conditions.

The value of the parameter *K*_ISCC_ was the initiation criteria for stress corrosion cracking. The measured *K*_ISCC_ values revealed the critical impact of stress intensity on the transformation of pitting in the weld area into cracking under certain corrosion environments and stress conditions. This also showed the influence of the growth depth of the pitting corrosion on stress corrosion behavior in the weld area. The change in *K* as a function of time is depicted in Figure 9. The DCPD measurements of crack propagation through a constant displacement CT specimen showed a decline in *K* to a certain value before achieving stability at the crack growth rate of less than 10^−8^ mm/s. The *K*_ISCC_ values in the weld area under normal and deviation water quality conditions were estimated to be 22.67 ± 0.25 MPa·m^1/2^ and 17.93 ± 0.17 MPa·m^1/2^, respectively. Therefore, the deviation water quality significantly decreased the *K*_ISCC_ value of the 20 steel/316L weld joint, indicating a greater SCC risk.

## 4. Discussion

### 4.1. Effect of Vortex Flow

The anodic dissolution reaction during the corrosion process of 20 steel can be described by the equations below:

(6) $$Fe⇌{Fe}^{2+}+2e^{-}$$

(7) $$Fe⇌{Fe}^{3+}+3e^{-}$$

In a steady-state aqueous solution, Fe^2+^ and Fe^3+^ might have accumulated at the interface between the metal and solution during the corrosion process. With the increase in Fe^2+^ and Fe^3+^ concentrations, the potential of the anodic reaction increased in a positive direction, and the corrosion was then suppressed. This meant that Fe^2+^ and Fe^3+^ cations just could have been diluted by the diffusion based on the concentration gradient from the metal surface to the solution. The cations could be taken away by the flow, especially the vortex flow, which provided a faster migration speed than the diffusion process in the steady state. The potential of the anodic reaction in the vortex flow was lower than that in the steady state. Therefore, the vortex significantly promoted the anodic dissolution of 20 steel in the pitting area, resulting in the increase in the depth and width of pitting. As earlier demonstrated [36,37], different locations of pipeline defects exhibited different mass transfer behaviors and corrosion changes.

The mass transfer could be significantly promoted by the turbulent vortex, and the corrosive H^+^ ions at the bottom of the pitting were quickly supplied from the fluid. In turn, the deposition of the protective corrosion product would be blocked by the turbulent vortex within the pitting area. The diffusion of ions in the normal and deviation water quality conditions was also accelerated, which induced the increase in the localized corrosion rate in the pitting area.

### 4.2. The Stress Corrosion Cracking Process

**Figure 10:**
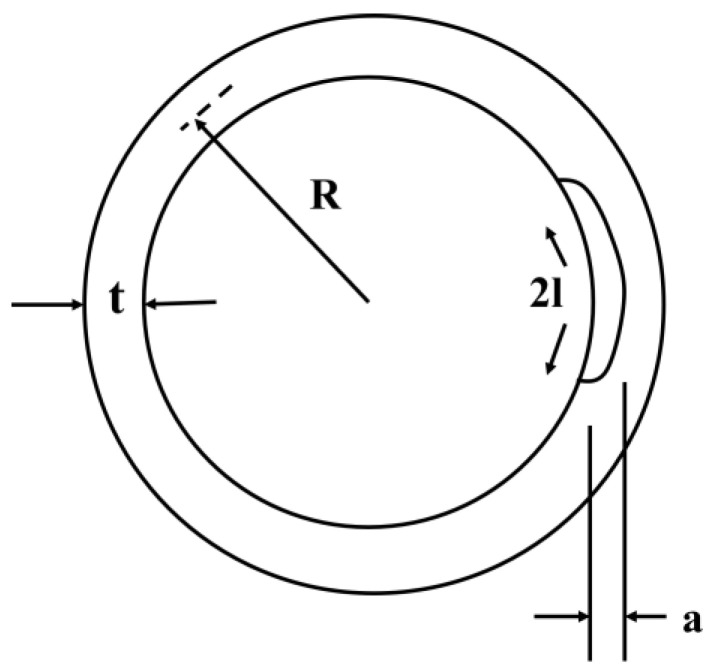
Internal surface defect structure diagram of the pipeline (here, t = 6 mm is the pipe wall thickness, R = 53 mm is the pipe radius, 2l is the crack width, and a is the crack depth).

**Figure 11:**
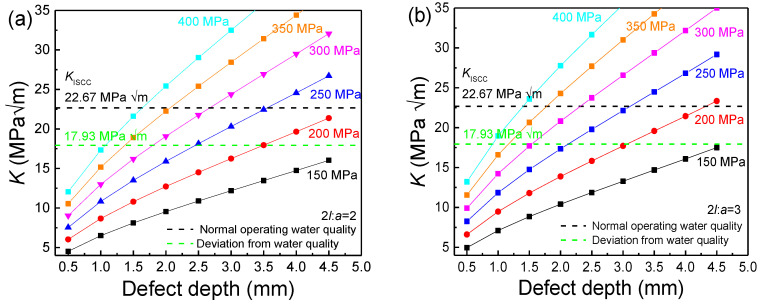

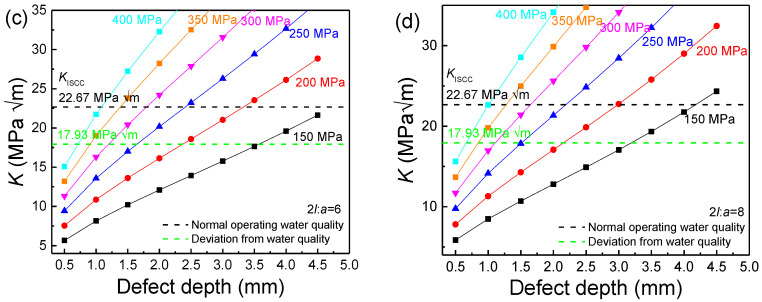
Defect depth dependence of *K* for the defect under different residual stress conditions. (**a**) 2*l*/*a* = 2; (**b**) 2*l*/*a* = 3; (**c**) 2*l*/*a* = 6; (**d**) 2*l*/*a* = 8.

Based on the actual pipeline size and defect structure, the *K* was calculated and is shown in Figure 10. The changes in the value of *K* in the four-point bending weld sample with different defect depths as a function of stress value, calculated using Equation (3), are shown in Figure 11. In the case of K exceeding *K*_1SCC_ under a specific environment, the stress corrosion crack initiated from the localized corrosion area rapidly grew until inducing pipeline failure. Meanwhile, the residual stress and width/depth ratio of the defects significantly affected the relationship between *K* and defect depth. At *K* exceeding *K*_ISCC_ under corresponding conditions, the cracking was quickly initiated, developing into stress corrosion cracking. Figure 11 reveals the effect of axial stress and defect depth on the changes in *K*. The value of *K* increased with the defect depth in the form of a parabolic curve at a specific residual stress. In the case of the width depth ratio of 2 (Figure 11a), the value of *K* was below the *K*_ISCC_ level at a residual stress of 150 MPa and a defect depth between 4 and 5 mm. At the width depth ratio of 2 and 200 MPa, the *K*_ISCC_ level could not be reached even at a defect depth exceeding 4 mm under normal operation water quality conditions, indicating a low risk of SCC. In turn, the value of *K* surpassed *K*_ISCC_ at a defect depth of about 3.5 mm in the case of deviation water quality, indicating that the SCC sensibility was promoted by deviation water quality. With the increase in residual stress, the value of *K* more easily achieved *K*_ISCC_.

**Figure 12:**
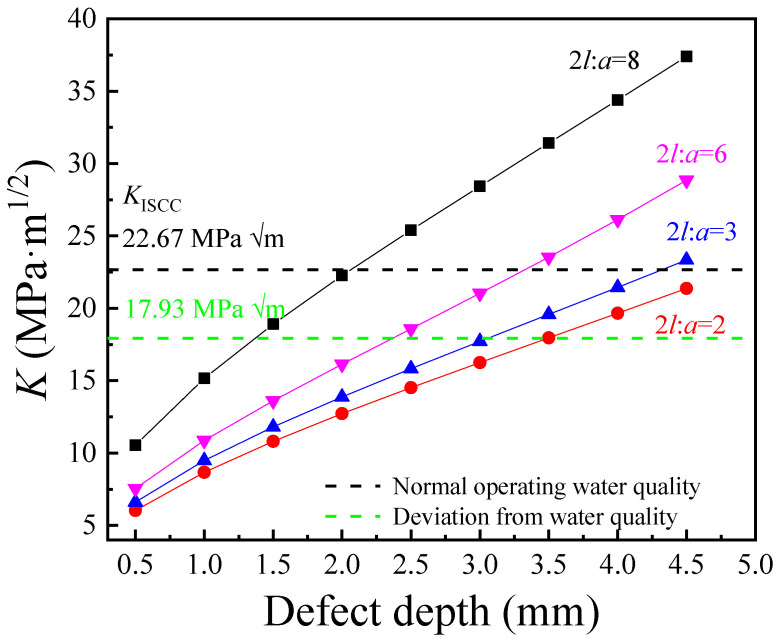
Defect depth dependence of *K* for the defect with different width/depth values.

Meanwhile, the increase in the width–depth ratio (2*l*/*a*) also increased the stress concentration at the defect, as shown in Figure 12. The curve showing the changes in *K* as a function of defect depth gradually downshifted with the increase in the 2*l*/*a* value, and *K* exceeded *K*_ISCC_ at a smaller defect depth. Therefore, the failure of the pipeline was closely related to the shape of the defect and the corrosion resistance of the weld area.

In general, the interaction of galvanic corrosion, residual stress, and flow velocity induced the initiation of SCC cracks from the localized defects in the weld area, resulting in the failure of the safe-end feedwater lines. The deviation water quality significantly promoted the localization and deterioration of SCC resistance within the weld area, especially for the 20 steel side. The *K*_ISCC_ value of the weld joint decreased under the deviation water quality conditions. The vortex in the pitting area further raised the pitting depth and the stress concentration increased, causing the value of *K* to easily reach the *K*_ISCC_ level.

## 5. Conclusions

The stress corrosion cracking failure of safe-end feedwater lines was studied via experimental lab testing and numerical simulation. Based on the findings, the following conclusions can be drawn.

(1) The failure of safe-end feedwater lines was controlled by the combined action of galvanic corrosion, residual stress, and flow. When the 20 steel was welded with 316L stainless steel, the 20 steel with low potential existed along with the galvanic corrosion. The obvious pitting corrosion and cracks could be observed on the 20 steel side of the weld joint. Combined with the field data, this was consistent with the hypotheses, and galvanic corrosion was indeed the critical failure factor.
(2) The vortex flow was detected around the welding bump and within the pits. The H^+^ ion concentration was shifted toward the upstream portion of the pitting. Then, the growth of the pits was accelerated in the horizontal direction, forming shallow-shaped pitting. Finally, the flow velocity promoted the pitting growth rate in both the vertical and horizontal directions. The pitting area provided stress concentration under residual stress conditions, which was consistent with the shape of the pitting observed. Therefore, it can be assumed that vortex flow existed in the safe-end feedwater lines.
(3) The increase in the width–depth ratio (2*l*/*a*) enhanced the stress concentration at the defect, with *K* exceeding *K*_ISCC_ at a smaller defect depth and the quick transformation of pitting into a crack. The deviation water quality significantly reduced the *K*_ISCC_ value of the weld joint. The stress corrosion cracking was initiated from the localized corrosion area and propagated along with the failures of the safe-end feedwater lines.

## Figures and Tables

**Table 1:** Chemical composition of experimental materials (mass fraction, percent).

Steel	C	Si	Mn	S	P	Cr	Ni	Cu	Fe
20	0.220	0.260	0.570	0.019	0.098	0.019	0.006	0.006	Bal.
316L	0.018	0.220	1.420	0.002	0.023	17.200	12.950	0.033	Bal.
316L welding wire	0.022	0.420	1.890	0.002	0.002	19.120	12.620	0.340	Bal.

**Table 2:** Water quality conditions of test solutions.

Environmental Parameter	Water Quality	
	Normal Operation Water Quality	Deviation Water Quality
Oxygen content (mg/L)	≤0.007	≤0.1
pH (25 °C)	7~8	9.6~10.3
Cl^−^ (mg/L)	≤0.1	≤5
Pressure (MPa)	2.6~3.2	2.6~3.2
Temperature (°C)	105	~180
Conductivity (μS/cm)	~50	~1600
H_2_PO^4−^/PO_4_^3−^ (mg/L)	0.1	30
SO_3_^2−^ (mg/L)	0.5	150

**Table 3:** Corrosion rates of 20 steel and 20 steel in the weld couple under normal operating water quality and deviation water quality conditions.

Conditions	Corrosion Rate (Mm/Yr)	
	20 Steel	20 Steel at Weld Couple
Normal operating water quality	0.053 ± 0.012	0.061 ± 0.009
Deviation water quality	0.39 ± 0.02	0.48 ± 0.03

**Table 4:** Residual stress of the weld area at the inner and outer surfaces.

	Residual Stress (MPa)	
	Inner Surface	Outer Surface
Sample 1	149.1 ± 11.7	−126.7 ± 30.3
Sample 2	156.9 ± 21.3	−169.1 ± 45.3
Sample 3	150.5 ± 17.9	−159.1 ± 44.4

## Data Availability

Data are contained within the article.
